# Early life fecal microbiota transplantation enhances fermentation potential by changing the microbial profiles in broiler chickens

**DOI:** 10.1016/j.psj.2025.106189

**Published:** 2025-12-02

**Authors:** Haoran Zhao, Muhammad Zeeshan Akram, Luke Comer, Matthias Corion, Elena Fako, Nadia Everaert

**Affiliations:** Nutrition and Animal-Microbiota Ecosystems Laboratory, Department of Biosystems, KU Leuven, Heverlee, Belgium

**Keywords:** Broiler, Citrus pectin, Fecal microbiota transplantation, Inulin, In vitro fermentation

## Abstract

The early gut microbiota of broiler chickens plays a critical role in shaping physiological functions later in life. Broilers have a limited capacity to utilize dietary fiber at an early stage of life. Fecal microbiota transplantation (**FMT**) can modify the gut microbial composition of broilers, potentially enhancing their fiber utilization capability. In this study, fecal samples from different chicken donors (broilers, laying hens, and broiler breeders) were collected and used for *in vitro* fermentation with two structurally distinct fibers, inulin and citrus pectin. FMT was then performed on newly hatched broilers, followed by additional *in vitro* fermentation to evaluate changes in the recipients’ fiber fermentation capacity. Laying hen fecal microbiota exhibited the fastest fermentation rates for both fibers, while broilers showed the slowest. Notably, laying hens produced the highest levels of propionic acid during fermentation. These donor-specific fermentation differences were likely driven by *Bacteroides, Subdoligranulum, Collinsella, Clostridium*, and *Bifidobacterium*. The *in vivo* experiment demonstrated that FMT significantly altered the microbial composition and volatile fatty acid production in recipient broilers up to 14 days of age. Subsequent *in vitro* fermentation of the recipients’ cecal content revealed that fermentation capacity was influenced by both the donor microbiota and the fiber substrate, with recipients of laying hen microbiota showing significantly enhanced propionic acid production, mirroring donor patterns. In conclusion, differences in donor fecal microbiota composition reflect their distinct capacities to utilize different fibers. Through FMT, recipient’s cecal microbiota composition can be changed, and the donor’s fermentative capacity is reflected in the recipients. These findings highlight the potential of early microbial interventions to improve fiber utilization in broilers, offering a promising strategy to optimize gut health.

## Introduction

The chicken gastrointestinal tract harbors a diverse and complex microbial community that plays a vital role in host nutrition metabolism (Rychlik, 2020). These microbes degrade dietary components indigestible to the host, particularly complex carbohydrates like non-starch polysaccharides, fermenting them into short-chain fatty acids (**SCFA**) (Mahmood and Guo, 2020). SCFA not only serve as an energy source for the host but also promote intestinal development and modulate immune function (Bedford and Gong, 2018; Liu et al., 2021). Additionally, the gut microbiota can ferment dietary fibers to synthesize micronutrients such as vitamins B and K (Mahmood and Guo, 2020). These metabolic functions are mediated by carbohydrate-active enzymes (**CAZymes**) secreted by the microbiota (Wardman et al., 2022). For instance, glycoside hydrolases and polysaccharide lyases produced by Bacteroidota efficiently break down plant-derived polysaccharides, providing fermentable carbon sources for microbial utilization (Wardman et al., 2022; Zechel and Withers, 2000). Notably, chicken gut microbiota composition and its metabolic outputs are shaped by host diet, breed, age, and management practices (Rychlik, 2020; Wardman et al., 2022). Specifically, laying hens harbor higher Bacteroidota abundances than broilers, a difference attributed to their fiber-rich diets, as this phylum encodes extensive CAZymes for polysaccharide breakdown (Videnska et al., 2014). Despite the recognized impact of these factors, little is known about how gut microbiota from distinct chicken donors differentially regulate fiber degradation, particularly in the context of microbial functional transfer.

Inulin is an oligosaccharide, usually composed of 2-60 fructose units and a terminal glucose (Tawfick et al., 2022). In contrast, citrus pectin is a more complex polysaccharide, primarily consisting of galactose, rhamnose, arabinose, and galacturonic acid polymers (Wu et al., 2021). Neither inulin nor citrus pectin can be digested by intestinal enzymes, but are instead primarily fermented by gut microbes into various metabolites (Tawfick et al., 2022; Wu et al., 2021). Key fiber-degrading bacteria from members of *Bacteroides* directly produce multiple glycoside hydrolases to metabolize inulin (Rakoff-Nahoum et al., 2016), while the degradation of more complex carbohydrates like pectin requires polysaccharide utilization loci (**PULs**) (Wardman et al., 2022). Interestingly, some microbes possess only partial PULs and may rely on cross-feeding interactions for complete pectin degradation (Wardman et al., 2022). However, it remains unclear how microbiota from different chicken donors utilize these structurally different fibers and whether fiber utilization efficiency can be modulated in the microbiota of newly hatched chicks.

In modern commercial poultry production, chicks lack a maternal microbial influence and primarily acquire microbes passively from the environment (Dai et al., 2022). This uncontrolled colonization often enables pathogens like *Escherichia coli* to dominate early gut niches due to their environmental abundance or competitive advantage, reshaping the intestinal environment to favor their survival and posing significant health risks (Rychlik, 2020; Wen et al., 2021). Given broilers’ short production cycle, early acquisition of optimal microbiota is crucial, as studies show that initial microbial colonization has long-term impacts on host health (Rychlik, 2020). The low microbial load in newly hatched chicks presents a critical window for microbiota modulation. Fecal microbiota transplantation (**FMT**) has recently been explored in poultry to introduce beneficial microbes, aiming to improve production performance, gut health, immunity, and lipid metabolism (Elokil et al., 2022; Gong et al., 2019; Ma et al., 2023; Metzler-Zebeli et al., 2019; Song et al., 2024). Notably, FMT may provide a solution to the long-standing challenge of dietary fiber utilization in poultry nutrition. Studies have reported negative correlations between fiber concentration and feed intake or nutrient digestibility in chickens (Mahmood and Guo, 2020). However, the nutritional value of dietary fiber is ultimately determined by microbial metabolic capacity (Mahmood and Guo, 2020; Wardman et al., 2022). To date, research investigating targeted microbial manipulation strategies to improve fiber utilization remains limited.

This study aims to: (1) compare *in vitro* fiber utilization patterns of gut microbiota from different chicken donors when exposed to two structurally distinct fibers (inulin and citrus pectin); (2) evaluate how FMT from these donors influences the microbiota composition of newly hatched chicks; and (3) assess the fermentation capacity in these chicks by repeating the *in vitro* fermentation after FMT. We focus on the influence of donor microbiota on the microbial composition and fermentation capacity of recipients, providing theoretical foundations for early-life gut microbiota modulation in broilers.

## Materials and methods

This animal study was approved by the Katholieke Universiteit Leuven Ethical Committee for Animal Experimentation (Ethical protocol P134/2023, Belgium) and conducted at the animal facility of the Department of Biosystems, KU Leuven, Belgium. All experiments were conducted in accordance with the necessary animal welfare and housing standards, following the recommendations of the ROSS 308 Broiler Management Manual, with experimental procedures reflective of ARRIVE guidelines.

### Donor selection

Feces were collected from three different donor sources with no history of antibiotic use: Ross 308 broilers, D 36 (broiler); ISA Brown laying hens, 30 weeks (laying hen); and Ross 308 parent broiler breeders, 35 weeks (broiler breeder). Fresh feces were collected from 10 birds per donor, with the white uric acid-rich portion of the excreta removed to minimize interference with the animal’s metabolism (Zhao et al., 2025). The collected feces were pooled and immediately transferred to the laboratory on ice to maintain sample integrity. The inoculum was prepared at a final concentration of 167 g/L by mixing feces with phosphate-buffered saline (**PBS**, pH = 7.4) at a 1:6 (w:v) ratio, following a standard protocol (Zhao et al., 2025). After vortexing, the mixture was centrifuged at 800 × *g* for 10 minutes. The supernatant was collected and filtered through a 200 µm sterile sieve (Retsch, Germany) to remove large particulate matter and ensure a uniform inoculum. The final inoculum was mixed with 10 % sterile glycerol and stored at −80°C for future use.

### Animals and housing

A total of 144 newly hatched male Ross 308 chicks were purchased from Belgabroed N.V. hatchery (Merksplas, Belgium) and selected as recipients. They were divided into four groups, each containing 36 chicks. The chicks were vaccinated *in ovo* against Newcastle disease and Gumboro on D 18 of incubation. During the first three days following hatching, the control group (CON) received 1 mL of saline once daily via oral gavage, while the FMT groups received 1 mL of their respective inocula once daily. The FMT groups were designated as FMT1 (receiving inoculum from Ross 308 broilers), FMT2 (receiving inoculum from ISA Brown laying hens), and FMT3 (receiving inoculum from Ross 308 broiler breeders). The housing temperature was maintained at 34°C initially and reduced by 1°C every two days, and chicks were reared on wood shavings. Lighting was provided for 23 hours during the first 10 days, followed by an 18-hour light and 6-hour dark cycle. The chicks had *ad libitum* access to water and standard feed (Supplementary file 1, Table S1) and were raised until D 14.

### Sample collection

On days 5, 7, and 14 post-hatching, ten birds from each group, selected based on average body weight, were euthanized for sampling. The chicks were subjected to electronarcosis followed by decapitation. Cecal contents were collected, snap-frozen, and stored at −80°C for further analysis.

### In vitro fermentation

Inulin and citrus pectin were selected as dietary fibers with distinct structural characteristics (Tawfick et al., 2022; Wu et al., 2021). Inulin (Fibruline Instant®) was provided by Cosucra Groupe Warcoing SA (Warcoing, Belgium). Citrus pectin was provided by Royal Agrifirm Group (Apeldoorn, The Netherlands). All other chemicals used were of analytical grade.

For the donor *in vitro* fermentation, the aforementioned fecal inocula were used. For the recipient *in vitro* fermentation, cecal content was obtained from the aforementioned recipients of D 14. For each group, cecal content from 10 birds was pooled in a 50 mL sterile tube, flushed with a gas mixture (90 % N_2_, 5 % H_2_, 5 % CO_2_), and placed on ice. The pooled cecal content was then diluted with PBS at a ratio of 1: 9 (w: v) and homogenized with a blender (Mixwel, Alliance Bio Expertise), and centrifuged at 800 × *g* for 10 minutes to prepare the inocola. The inocula were filtered through a 200 µm sieve (Retsch, Germany) and were then ready to use. The medium for *in vitro* batch fermentation was prepared as described by Uerlings et al. (2020) with small modifications. Briefly, the medium consisted of 1.35 g/L Na_2_HPO_4_, 1.47 g/L KH_2_PO_4_ as buffers, 0.14 g/L MgSO_4_·7H_2_O, 15.84 mg/L CaCl_2_·2H_2_O, 12 mg/L MnCl_2_·4H_2_O, 1.20 mg/L CoCl_2_·6H_2_O, 0.96 mg/L FeCl_3_·6H_2_O as essential minerals for microbial growth, 8.3 g/L NaHCO_3_, 0.98 g/L NH_4_HCO_3_ for pH stabilization and nitrogen supply, 0.3 g/L Na_2_S·9H_2_O as a reducing agent, and 1 mg/L resazurin as an oxygen indicator, with a final pH of 6.4. For the fermentation substrates, 0.1 g of inulin or citrus pectin was weighed in triplicate into 50 mL serum bottles. Subsequently, 2 mL of inoculum and 8 mL of medium were added, resulting in a fecal inoculum concentration of 4 % (w/v) for donor fermentation and 2 % (w/v) for recipient fermentation, with a dietary fiber concentration of 1 % (w/v) in both. Serum bottles without dietary fibers served as blank controls (*n* = 3 technical replicates). The bottles were flushed with N₂ for 10 seconds, sealed, and incubated in a water bath at 41°C with a stirring shaft speed of 80 rpm for 60 hours.

For each inoculum and substrate, six bottles were prepared per group: three technical replicates for gas measurements, followed up in time and three technical replicates per timepoint for volatile fatty acid (**VFA**), lactic acid (**LA)**, and microbiota measurements. For donor fermentation, samples were collected at 6, 12, and 24 hours to analyze VFA and LA, with microbiota analysis performed on samples collected at 12 hours, as this time point captures a representative microbial community during active fermentation. For recipient fermentation, only the VFA concentration was measured at 12 hours. The VFAs in fermentation broth and cecal content were measured according to Akram et al. (2024). The LA was measure according to Uerlings et al. (2020). Gas pressure was measured at 2, 5, 8, 12, 16, 20, 24, 36, 48, and 60 hours of fermentation (*n* = 3 technical replicates) using a digital pressure gauge (DG25, Ashcroft). The recorded gas pressure values (bar) were converted into cumulative gas volume (mL/g) and fitted using the mathematical monophasic model (Groot et al., 1996), which allowed the calculation of fermentation kinetics including the total gas production (**A**), the time to half asymptote (**B**), the maximum rate of gas production (**R_max_**), and the time at which the maximum rate of gas production is reached (**T_max_**). The fitted cumulative gas volume were then plotted against fermentation time, and the corresponding residuals were visualized and provided in Supplementary file 2 (Fig. S1 and Fig. S2).

### DNA extraction, 16 s rRNA sequencing, and bioinformatics workflow

DNA was extracted from the fermentation inoculum after 12 h of *in vitro* fermentation, the cecal contents of recipient broilers on D 5, D 7, and D 14, and the feces of donors using the QIAamp PowerFecal Pro DNA Kit (Qiagen Benelux B.V., Venlo, the Netherlands). The concentration and quality of the extracted DNA were measured using a Nanodrop 2000 spectrophotometer (Thermo Fisher Scientific, Waltham, MA). The V3-V4 regions of the 16S rRNA gene were amplified with barcoded primers (341F: CCTAYGGGRBGCASCAG; 806R: GGACTACNNGGGTATCTAAT). The libraries were sequenced on the Illumina NovaSeq 6000 platform by Novogene Co., Ltd (Germany) to generate 250 bp paired-end raw reads. The average sequencing depth was approximately 50 000 reads per sample (Supplementary file 2, Fig. S3). The raw sequencing was then filtered and demultiplexed on the QIIME2 platform (v2024.9). DADA2 was employed for noise reduction and the generation of amplicon sequence variants (**ASVs**). Rarefaction curves (Supplementary file 2, Fig. S4) indicated that the sequencing depth was sufficient to capture the microbial diversity of all samples. For alpha and beta diversity analyses, data was rarefied as recommend by Schloss (2024). This was performed to the depth of the sample with the least depth (43 507 reads for the *in vivo* experiment and 49 093 reads for the *in vitro* experiment). Taxonomic assignment of ASVs was performed using the SILVA database (version 138.1/2) with a 99 % sequence identity threshold using the Naive Bayes Classifier method (Akram et al., 2024; Robeson et al., 2021). Additionally, a mapping of feature IDs to sequences and a rooted phylogenetic tree were generated as artifacts in QIIME2 (v2024.9). Subsequently, these artifacts were imported into RStudio (V4.2.3) for further analysis.

### Statistical analysis

*In vitro* fermentation kinetics parameters, SCFA, and LA produced from *in vitro* fermentation, alpha diversity, and microbial phylum-level relative abundance were analyzed using Kruskal-Wallis (**KW**) test followed by Dunn’s post hoc test. Concentrations of VFA in cecal content were using one-way ANOVA followed by Tukey’s post hoc test. Microbial functional predictions were performed using PICRUSt2 and utilized the MetaCyc Metabolic Pathway Database as a reference. Microbial functional predictions, derived from PICRUSt2, were also compared using the KW test with Dunn’s post hoc analysis, and the top 10 pathways with the lowest adjusted *P*-values were selected for visualization. Beta diversity of microbial communities and PICRUSt2-derived functional predictions was assessed using principal coordinates analysis (**PCoA**) based on Bray-Curtis distances, with statistical significance determined by PERMANOVA. Pairwise PERMANOVA comparisons were conducted to identify specific group differences. All *P*-values were adjusted for multiple comparisons using the Benjamini-Hochberg false discovery rate (**FDR**) method, with statistical significance defined as an adjusted *P*-value <0.05. Differentially abundant genera were identified using LEfSe analysis implemented in R with the microbiomeMarker package, using a Kruskal-Wallis *P*-value cutoff of 0.05, and opting for a stringent linear discriminant analysis score (LDA) threshold of |LDA| > 4 to select the most significant results. The Kruskal-Wallis *P*-values from the LEfSe output were exported and adjusted for multiple comparisons using the FDR method. Genera with adjusted *P* < 0.05 and |LDA| > 4 were retained for downstream visualization and analysis. Spearman correlation analysis was subsequently performed between the LEfSe-identified genera and cecal VFA concentrations to assess their associations.

## Results

### Microbiota composition of donor feces

**Fig. 1:**
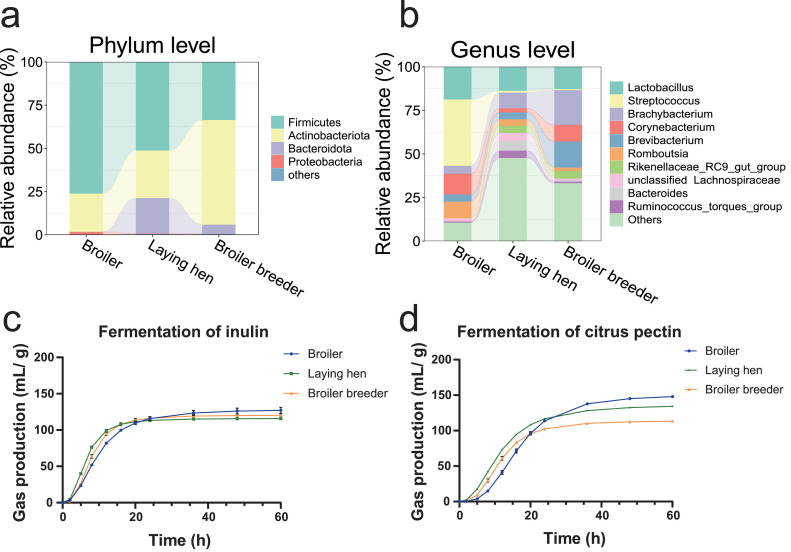
Composition of fecal microbiota and fitted cumulative gas production profiles during *in vitro* donor fermentation. **(a)** Phylum-level taxonomic composition of donor fecal microbiota, displaying mean relative abundance (%). **(b)** Genus-level taxonomic composition of donor fecal microbiota, displaying mean relative abundance (%). **(c)** Fitted cumulative gas production kinetics during *in vitro* donor fermentation of inulin (mL/g). **(d)** Fitted cumulative gas production kinetics during *in vitro* donor fermentation of citrus pectin (mL/g). Incubation temperature during fermentation: 41°C. Data represent mean values ± SEM. *n* = 3 technical replicates.

As shown in Fig. 1 (a, b), the fecal microbiota composition differed among the three donor types. In broiler samples, the community was dominated by phylum Firmicutes (76.2 %) and Actinobacteriota (22.1 %), with *Streptococcus* (38.1 %) and *Lactobacillus* (18.8 %) as the most abundant genera, followed by *Corynebacterium* (11.9 %). In contrast, laying hens exhibited a more diverse microbial profile, characterized by phylum Firmicutes (51.3 %), Actinobacteriota (27.5 %), and Bacteroidota (20.5 %). Among genera, *Lactobacillus* (13.8 %) remained prevalent, followed by *Brachybacterium* (8.9 %) and *Bacteroides* (5.6 %). The microbiota of broiler breeders was composed of phylum Actinobacteriota (60.5 %) and Firmicutes (33.6 %), with genera including *Brachybacterium* (19.9 %), *Brevibacterium* (15.0 %), and *Lactobacillus* (12.9 %).

### Donors drive distinct fermentation profiles of inulin and citrus pectin

To assess the function of the gut microbiota from different donors, we performed *in vitro* batch fermentations of both inulin and citrus pectin. As shown in Fig. 1 (c, d) and Table 1, no significant difference between donor types was observed in total gas production (**A**) for inulin (*P* = 0.057), whereas broilers exhibited the highest A, followed by laying hens and broiler breeders for citrus pectin (*P* < 0.001). Laying hens exhibited the most rapid fermentation for both substrates, with a higher maximum rate of gas production (**R_max_**), a shorter time to half asymptote (**B**), and a faster time to reach the maximum rate of gas production (**T_max_**; *P* < 0.001). In contrast, broilers showed the slowest fermentation, with the longest B and T_max_ (*P* < 0.001).

**Table 1:** *In vitro* fermentation kinetics of inulin and citrus pectin by donor types.

Fermentation of inulin					
	A (mL/g DM)	B (h)	R_max_ (mL/g DM/h)	T_max_ (h)	R^2^
Broiler	128.87	9.47^a^	9.67^c^	6.43^a^	0.9922
Laying hen	116.02	6.31^c^	14.57^a^	4.81^c^	0.9983
Broiler breeder	120.50	7.69^b^	12.91^b^	6.02^b^	0.9953
SEM	2.40	0.46	0.73	0.25	
*P* value	0.057	<0.001	<0.001	<0.001	
Fermentation of citrus pectin					
Broiler	150.87^a^	16.60^a^	7.72^b^	13.26^a^	0.9581
Laying hen	136.96^b^	11.33^b^	8.52^a^	7.64^c^	0.9694
Broiler breeder	114.04^c^	11.43^b^	8.26^a^	8.98^b^	0.9641
SEM	5.43	0.88	0.13	0.86	
*P* value	<0.001	<0.001	0.006	<0.001	

Note: The different letters in a row mean a significant difference (*P* < 0.05). *n* = 3 technical replicates. A: the total gas production. B: the time to half asymptote. R_max_: the maximum rate of gas production. T_max_: the time at which the maximum rate of gas production is reached. DM: dry matter. SEM: Standard error of the mean. R^2^: coefficient of determination.

As shown in Table 2, during inulin fermentation, laying hens consistently produced the highest acetic and propionic acid concentrations at all timepoints (*P* < 0.001). Butyric acid concentrations were higher in laying hens at 6 and 12 h (*P* < 0.001), while at 24 h, broiler breeders exhibited similar butyric acid levels to laying hens but higher than broilers (*P* < 0.001). Lactic acid concentrations showed no significant differences among donors at 6 and 24 h (*P* > 0.05), but at 12 h, laying hens and broiler breeders produced higher lactic acid than broilers (*P* < 0.001).

**Table 2:** Short-chain fatty acid and lactic acid concentrations produced during *in vitro* fermentation of inulin and citrus pectin by donor types (mM).

Fermentation of inulin				
	Acetic acid	Propionic acid	Butyric acid	Lactic acid
6h				
Broiler	0.15^c^	0.00^b^	0.00^b^	20.91
Laying hen	0.41^a^	0.05^a^	0.02^a^	24.48
Broiler breeder	0.18^b^	0.00^b^	0.00^b^	20.62
SEM	0.04	0.01	<0.01	0.80
*P* value	<0.001	<0.001	<0.001	0.061
12h				
Broiler	0.33^b^	0.00^c^	0.00^c^	38.30^b^
Laying hen	0.90^a^	0.15^a^	0.08^a^	57.41^a^
Broiler breeder	0.46^b^	0.04^b^	0.03^b^	51.92^a^
SEM	0.09	0.02	0.01	3.00
*P* value	<0.001	<0.001	<0.001	<0.001
24h				
Broiler	0.45^c^	0.00^c^	0.04^b^	62.63
Laying hen	1.35^a^	0.17^a^	0.14^a^	60.46
Broiler breeder	0.89^b^	0.09^b^	0.16^a^	62.76
SEM	0.13	0.03	0.02	0.72
*P* value	<0.001	<0.001	<0.001	0.396
Fermentation of citrus pectin				
6h				
Broiler	0.23^b^	0.00^b^	0.00	13.89
Laying hen	0.43^a^	0.09^a^	0.00	13.06
Broiler breeder	0.22^b^	0.00^b^	0.00	13.00
SEM	0.03	0.02	0.00	0.22
*P* value	<0.001	<0.001	NA	0.194
12h				
Broiler	0.45^c^	0.00^c^	0.00^b^	15.93^a^
Laying hen	0.96^a^	0.26^a^	0.09^a^	14.00^b^
Broiler breeder	0.71^b^	0.10^b^	0.00^b^	13.29^b^
SEM	0.07	0.04	0.01	0.42
*P* value	<0.001	<0.001	<0.001	0.002
24h				
Broiler	1.22^b^	0.09^c^	0.12^c^	18.09
Laying hen	1.61^a^	0.91^a^	0.83^b^	18.36
Broiler breeder	1.17^b^	0.49^b^	1.00^a^	17.28
SEM	0.07	0.12	0.14	0.35
*P* value	<0.001	<0.001	<0.001	0.491

Note: The different letters in a row mean a significant difference (*P* < 0.05). *n* = 3 technical replicates. SEM: Standard error of the mean. NA: not available.

During citrus pectin fermentation (Table 2), laying hens also produced the highest acetic and propionic acid concentrations at all timepoints (*P* < 0.001). No butyric acid was detected at 6 h, but at 12 h, laying hens yielded higher butyric acid concentrations than broilers and broiler breeders (*P* < 0.001), with levels increasing further at 24 h, where laying hens and broiler breeders showed higher concentrations than broilers (*P* < 0.001). Lactic acid concentrations were not significantly different among donors at 6 and 24 h (*P* > 0.05), but at 12 h, broilers produced the highest lactic acid concentrations (*P* < 0.001).

### Donors drive distinct microbiota shifts in the fermentation of inulin and citrus pectin

**Fig. 2:**
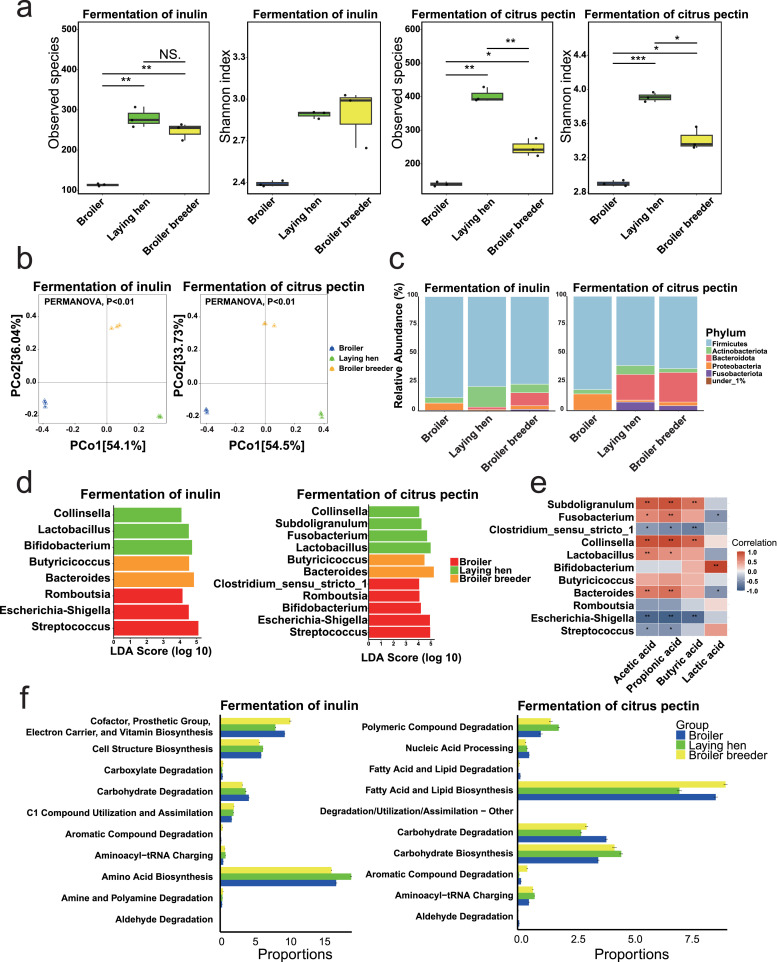
Donor-specific microbial profiles after 12 h of *in vitro* fermentation. **(a)** Alpha diversity (Observed species and Shannon index) after 12 h of fermentation. **(b)** Principal coordinates analysis of beta diversity based on Bray-Curtis dissimilarity at 12 h. **(c)** Phylum-level taxonomic composition after 12 h fermentation (%). **(d)** Linear discriminant analysis Effect Size (**LEfSe**) analysis identifying differentially abundant genera. **(e)** Spearman correlation heatmap between short-chain fatty acids, lactic acid, and LEfSe-identified genera. **(f)** Top 10 enriched MetaCyc pathways predicted by PICRUSt2, ranked by FDR-adjusted *P* values. *n* = 3 technical replicates. Statistical analyses: Kruskal-Wallis test with Dunn’s post hoc test for alpha diversity and predicted functional pathway; PERMANOVA for beta diversity; LEfSe for differential genera with |LDA| > 4 and FDR-adjusted *P* < 0.05; Spearman correlation for metabolite–genus associations (red = positive, blue = negative); significance indicated as ***P < 0.001, **P < 0.01, *P < 0.05, NS: not significant.

We determined the microbiota composition at 12 h of fermentation. As shown in Fig. 2a, during inulin fermentation, laying hens and broiler breeders exhibited significantly higher alpha-diversity in terms of observed species compared to broilers (*P* < 0.05), while the Shannon index showed no significant difference (*P* > 0.05). On the other hand, during citrus pectin fermentation, laying hens displayed significantly higher observed species and Shannon index values compared to broilers and broiler breeders (*P* < 0.05). Beta-diversity analysis revealed significant differences in microbial community structure during inulin and citrus pectin fermentation, with distinct clustering by donor type (Fig. 2b, *P* < 0.05).

Microbial composition at the phylum level during fermentation varied significantly by donor type (Fig. 2c). In inulin fermentation, Firmicutes dominated all donor groups, but the second-most abundant phylum differed: Proteobacteria (6.2 %) in broilers, Actinobacteriota (18.0 %) in laying hens, and Bacteroidota (11.4 %) in broiler breeders. A similar Firmicutes dominance pattern was observed during citrus pectin fermentation. While broilers’ and broiler breeder’s microbiota maintained comparable phylum profiles between both substrates, laying hens exhibited a notable shift: Bacteroidota (22.5 %) instead of Actinobacteriota (7.8 %) as the second most abundant phylum during citrus pectin fermentation. At genus level (Supplementary file 1, Table S2), *Enterococcus* dominated across donors, followed by *Streptococcus* (21.7 %) in broilers, *Bifidobacterium* (11.9 %) and *Lactobacillus* (11.1 %) in laying hens, and *Bacteroides* (10.8 %) in broiler breeders in the fermentation of inulin. Citrus pectin fermentation by different donors also resulted in distinct microbial composition shifts at the genus level. *Enterococcus* remained dominant in broilers (42.5 %) and broiler breeders (42.2 %), while *Enterococcus* (22.6 %)*, Lactobacillus* (23.7 %) and *Bacteroides* (20.5 %) were more evenly represented in laying hens.

LEfSe analysis further revealed distinct donor-specific microbial signatures following inulin and citrus pectin fermentation (Fig. 2d). During inulin fermentation, broilers’ microbiota showed significant enrichment of *Streptococcus, Escherichia-Shigella*, and *Romboutsia*, whereas broiler breeders’ microbiota exhibited higher abundances of *Bacteroides* and *Butyricicoccus*. The laying hens’ microbiota was characterized by elevated *Lactobacillus, Bifidobacterium*, and *Collinsella*. For citrus pectin fermentation, broiler breeders maintained similar enrichment patterns as observed with inulin. The broilers’ microbiota demonstrated additional enrichment in *Bifidobacterium* and *Clostridium_sensu_stricto_1*. In contrast, laying hens showed no *Bifidobacterium* enrichment compared to inulin fermentation but was uniquely associated with *Lactobacillus* and *Collinsella*, along with newly enriched *Subdoligranulum* and *Fusobacterium*.

Spearman correlation analysis identified significant associations between microbial genera and fermentation metabolites (Fig. 2e). *Subdoligranulum, Fusobacterium, Collinsella, Lactobacillus*, and *Bacteroides* showed positive correlations with acetic acid and propionic acid. *Subdoligranulum* and *Collinsella* also exhibited a positive correlation with butyric acid, while *Bifidobacterium* was positively correlated with lactic acid.

As shown in Fig. 2f, the top 10 metabolic pathways with the smallest *P*-values were identified, highlighting the functional differences among microbiota from different chicken donors during fiber fermentation. During inulin fermentation, broiler breeders exhibited an elevated potential for cofactor, vitamin, and electron carrier biosynthesis. In contrast, laying hens showed the highest proportion of amino acid biosynthesis, while broilers exhibited higher proportions of carbohydrate degradation. For citrus pectin fermentation, the function of fatty acid and lipid biosynthesis was enriched in the broiler breeders group. Laying hens exhibited higher activity in carbohydrate biosynthesis, while carbohydrate degradation was enriched in broilers.

### FMT alters the VFA composition of recipient’s cecal contents

**Fig. 3:**
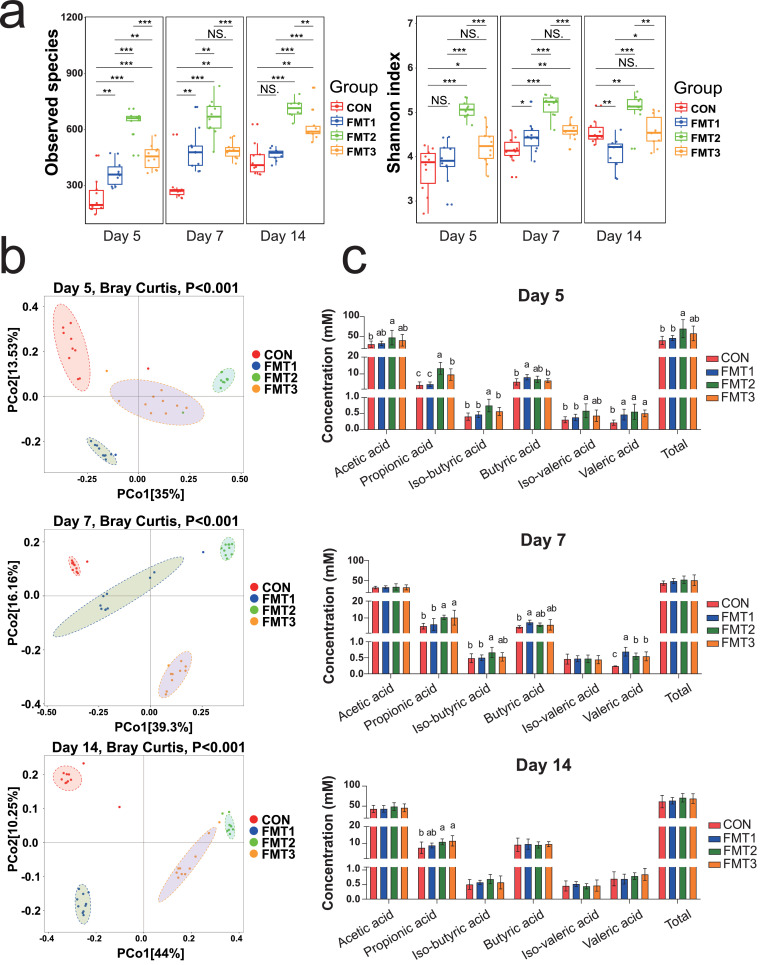
Microbial and metabolic profiles in cecal content of recipients following FMT at three timepoints. **(a)** Alpha diversity (Observed species and Shannon index) of recipient cecal microbiota. **(b)** Principal coordinates analysis of beta diversity based on Bray-Curtis dissimilarity in cecal content of recipient at three timepoints. **(c)** Cecal volatile acid concentrations across three timepoints (mM). FMT1, FMT2, and FMT3 received inocula from broilers, laying hens, and broiler breeders, respectively. *n* = 10 biological replicates. Statistical analyses: Kruskal-Wallis test with Dunn’s post hoc test for alpha diversity; PERMANOVA for beta diversity; one-way ANOVA with Tukey’s post hoc test for volatile acid concentrations; significance indicated as ***P < 0.001, **P < 0.01, *P < 0.05, NS: not significant.

On D 5, the concentrations of acetic acid and iso-valeric acid in cecal content of FMT2 was significantly higher than CON. The concentrations of propionic acid and iso-butyric acid in FMT2 and FMT3 were higher than CON (Fig. 3c, *P* < 0.05). The concentrations of butyric acid in FMT1 were higher than CON (*P* < 0.05). The concentrations of valeric acid were higher in all the FMT groups compared to the CON group (*P* < 0.05). The total VFA concentrations in FMT2 was higher than CON (*P* < 0.05).

On D 7, the concentrations of propionic acid in FMT2 and FMT3 were higher than CON, while the concentrations of iso-butyric acid in FMT2 were higher than CON (*P* < 0.05). The concentrations of butyric acid in FMT1 were higher than CON (*P* < 0.05). Again, the concentrations of valeric acid were higher in all the FMT groups compared to the CON group (*P* < 0.05), whereas other SCFA concentrations did not differ significantly (*P* > 0.05).

On D 14, the concentrations of propionic acid in FMT2 and FMT3 were higher than CON (*P* < 0.05), whereas other SCFA concentrations did not differ significantly (*P* > 0.05).

### FMT alters the microbial composition of the recipient’s cecum

***Alpha Diversity.*** On D 5 and D 7, the observed species of all the FMT groups was higher than CON (Fig. 3a, *P* < 0.05). The Shannon index of FMT2 and FMT3 were higher than CON (*P* < 0.05). The Shannon index of FMT1 is higher than CON on D 7, whereas there were no differences between FMT1 and CON (*P* > 0.05) on D 5.

On D 14, the observed species of FMT2 and FMT3 were higher than CON (*P* < 0.05), whereas there were no differences between FMT1 and CON (*P* > 0.05). The Shannon index of FMT1 was lower than CON, but FMT2 was higher than CON (*P* < 0.05), and there was no difference between FMT3 and CON (*P* > 0.05).

***Beta Diversity.*** PCoA based on Bray-Curtis distances revealed the beta-diversity of microbial communities across treatment groups at D 5, 7, and 14 (Fig. 3b). For each timepoints, all the groups formed a distinct cluster (*P* < 0.05).

**Fig. 4:**
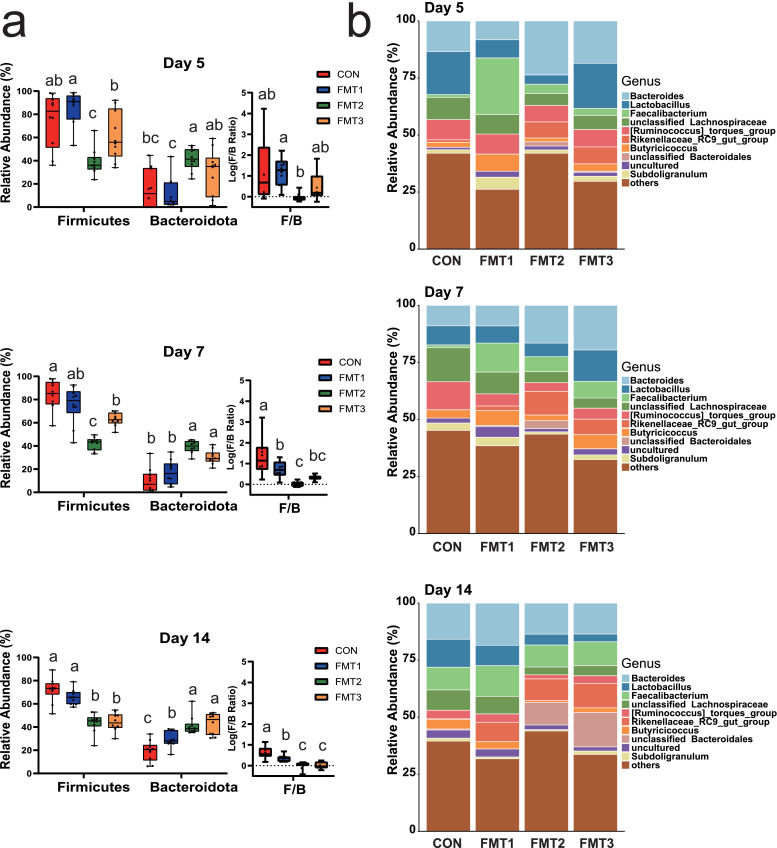
Temporal changes in cecal microbiota composition in recipient. **(a)** Relative abundances of Firmicutes and Bacteroidota, and the Firmicutes-to-Bacteroidota ratio (**F/B**, presented as log10 (F/B)) across three timepoints. **(b)** Genus-level taxonomic composition of recipient cecal microbiota, displaying mean relative abundance (%). FMT1, FMT2, and FMT3 received inocula from broilers, laying hens, and broiler breeders, respectively. *n* = 10 biological replicates. Statistical analyses: Kruskal-Wallis test with Dunn’s post hoc test for relative abundance and F/B ratio; different letters indicate significant differences (*P* < 0.05).

***Microbiota Composition.*** Microbiota composition showed a significant shift over time (Fig. 4; Supplementary file 1, Table S3.1). On D 5 and D 7, the compositions of the gut microbiota in CON and FMT1 had high relative abundances of Firmicutes (75.1 % to 85.8 %), with minor contributions from Bacteroidota (9.9 % to 17.1 %). However, the Firmicutes (38.9 % to 42.1 %) and Bacteroidota (39.3 % to 41.5 %) showed comparable relative abundances in FMT2. The Firmicutes (61.5 % to 63.0 %) also dominated in FMT3 but less so than in CON and FMT1, however Bacteroidota (30.3 % to 30.0 %) was higher than CON and FMT1 but lower than FMT2. It is also worth noting that Actinobacteriota exhibited higher relative abundance in the FMT2 group on D 5 and D 7 (7.0 % to 9.9 %). On D 14, The Firmicutes (72.0 %) in CON still dominated. The Bacteroidota increased slightly to 29.3 % in FMT1. The Firmicutes (43.7 % to 44.4 %) and Bacteroidota (41.2 % to 42.7 %) showed comparable relative abundances in FMT2 and FMT3. The Firmicutes-to-Bacteroidota ratio (F/B), expressed as log(F/B), was significantly lower in FMT2 and FMT3 compared to the CON on D7 and D14 (Fig. 4a, *P* < 0.05).

At the genus level (Fig. 4b; Supplementary file 1, Table S3.2), CON exhibited a predominance of *Lactobacillus* (18.9 %) and *Bacteroides* (13.4 %) on D 5, followed by unclassified *Lachnospiraceae* (9.7 %), and the *[Ruminococcus]_torques*_group (8.9 %). FMT1 displayed high abundance of *Faecalibacterium* (24.8 %), followed by the *[Ruminococcus]_torques*_group (8.8 %), unclassified *Lachnospiraceae* (8.6 %), *Bacteroides* (8.3 %). FMT2 has high abundance of *Bacteroides* (23.7 %), followed by *[Ruminococcus]_torques*_group (7.3 %), and *Rikenellaceae _RC9_gut_group* (7.0 %). FMT3 exhibited high abundance of *Lactobacillus* (19.7 %) and *Bacteroides* (18.6 %).

On D 7, unclassified *Lachnospiraceae* (14.9 %) and the *[Ruminococcus]_torques*_group (12.3 %) were the most abundant genera in CON. *Faecalibacterium* (12.7 %) remained the most abundant genus in FMT1, followed by unclassified *Lachnospiraceae* (9.6 %), and *Bacteroides* (9.0 %). In FMT2, *Bacteroides* (16.6 %) dominated, followed by the *Rikenellaceae_RC9_gut_group* (10.4 %). In FMT3, *Bacteroides* (19.6 %) and *Lactobacillus* (13.7 %) were the two most abundant genera.

On D 14, *Bacteroides* showed the highest abundance in CON, FMT1, and FMT2 (15.8 %, 18.5 %, 13.6 %, respectively), whereas unclassified *Bacteroidales* (15.1 %) was the most abundant in FMT3. The second most abundant genus in all the groups was *Lactobacillus* (12.3 %), followed by *Faecalibacterium* (13.4 %), unclassified *Bacteroidales* (9.9 %), and *Bacteroides* (13.6 %), respectively.

**Fig. 5:**
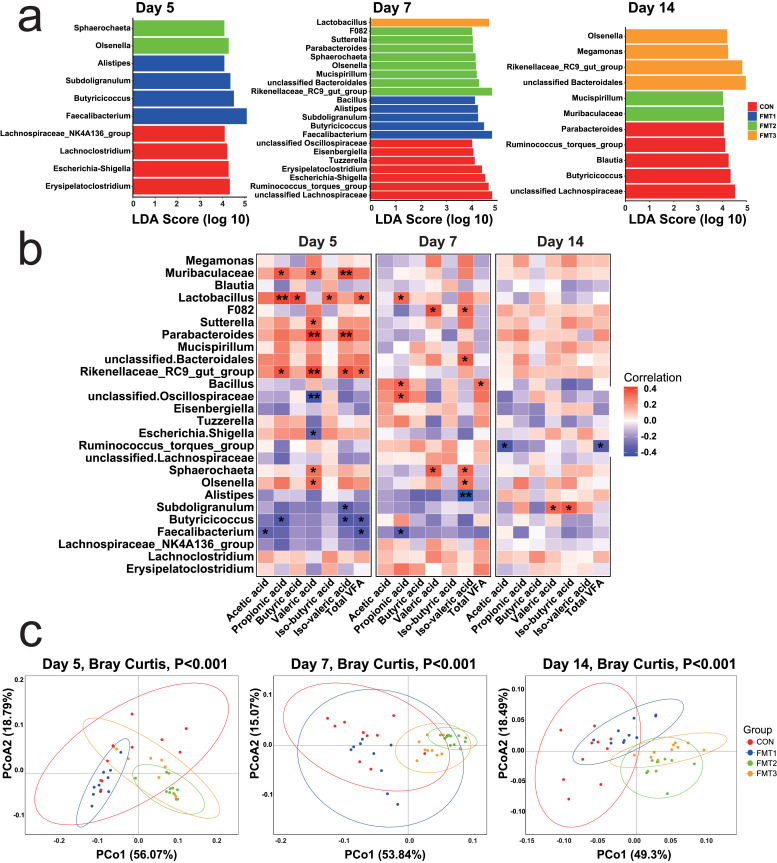
Microbial and functional alterations in cecal content of recipients across three timepoints. **(a)** Linear discriminant analysis Effect Size (**LEfSe**) analysis identifying differentially abundant genera in recipient microbiota. **(b)** Spearman correlation heatmap between LEfSe-identified genera and cecal volatile fatty acid (**VFA**) concentrations. **(c)** Principal coordinates analysis of predicted microbial functions (MetaCyc pathways) based on Bray-Curtis dissimilarity. FMT1, FMT2, and FMT3 received inocula from broilers, laying hens, and broiler breeders, respectively. *n* = 10 biological replicates. Statistical analyses: LEfSe for differential genera with |LDA| > 4 and FDR-adjusted *P* < 0.05; Spearman correlation for metabolite–genus associations (red = positive, blue = negative, *P < 0.05, **P < 0.01); PERMANOVA for predicted microbial functions.

***Differential Abundance Analysis.*** On D 5, LEfSe analysis identified 10 differentially abundant genera across all groups (Fig. 5a). The CON exhibited enrichment of *Erysipelatoclostridium, Escherichia-Shigella, Lachnoclostridium, Lachnospiraceae_NK4A136_group*. FMT1 was enriched in *Faecalibacterium, Butyricicoccus, Subdoligranulum*, and *Alistipes* while FMT2 exhibited high enrichment of *Olsenella, Sphaerochaeta*.

On D 7, the analysis revealed 21 bacterial genera exhibiting differential abundance among all groups (Fig. 5a). The CON demonstrated enrichment of the *Ruminococcus_torques_group, Escherichia-Shigella, Erysipelatoclostridium, Tuzzerella, Eisenbergiella*, unclassified *Oscillospiraceae*, unclassified *Lachnospiraceae*. FMT1 showed higher *Faecalibacterium, Butyricicoccus, Subdoligranulum, Alistipes*, and *Bacillus*. FMT2 had a greater abundance of *Rikenellaceae_RC9_gut_group*, unclassified *Bacteroidales, Mucispirillum, Olsenella, Sphaerochaeta, Parabacteroides, Sutterella, F082*, while FMT3 had increased *Lactobacillus* abundances.

On D 14, the analysis identified 11 differentially abundant genera (Fig. 5a). CON showed enrichment of *Butyricicoccus, Blautia, Ruminococcus_torques_group, Parabacteroides*, and unclassified *Lachnospiraceae*. FMT2 exhibited higher abundance of *Muribaculaceae*, and *Mucispirillum*. FMT3 had a higher abundance of *Rikenellaceae_RC9_gut_group, Megamonas, Olsenella*, and unclassified *Bacteroidales*.

***Correlation Between LEfSe-identified Genera and Volatile Fatty Acids.*** *Muribaculaceae* showed positive correlations with propionic acid, valeric acid, and iso-valeric acid on D 5 (Fig. 5b). *Lactobacillus* was positively correlated with propionic acid, butyric acid, iso-butyric acid on D 5 and propionic acid on D 7. *Sutterella* was positively correlated with valeric acid, and *Parabacteroides* with valeric acid and iso-valeric acid. *Rikenellaceae_RC9_gut_group* positively correlated with propionic acid, valeric acid, and iso-valeric acid on D 5. *Sphaerochaeta* was positively correlated with valeric acid on D 5 and valeric acid and iso-valeric acid on D 7. *Olsenella* was positively correlated with valeric acid on D 5, and iso-valeric acid on D 7. *F082* was positively correlated with valeric and iso-valeric acid on D 7. Unclassified *Bacteroidales* was positively correlated with iso-valeric acid on D 7. *Bacillus* and unclassified *Oscillospiraceae* was positively correlated with propionic acid on D 7. *Subdoligranulum* was positively corelated with butyric and iso-valeric acid on D 14. In contrast, unclassified *Oscillospiraceae* and *Escherichia-Shigella* was negatively correlated with valeric acid on D 5. *Subdoligranulum* was negatively correlated with iso-valeric acid. *Butyricicoccus* was negatively correlated with propionic acid and iso-valeric acid on D 5. *Faecalibacterium* was negatively corelated with acetic acid on D 5 and propionic acid on D 7. *Alistipes* was negatively corelated with iso-valeric acid on D 7. *Ruminococcus_torques_group* was negatively corelated with acetic acid on D 14.

***Microbial Functional Prediction.*** PCoA of metabolic pathways associated with cecal microbiota revealed distinct clustering patterns (Fig. 5c; Supplementary 1, Table S4). On D 5, although FMT3 and CON did not differ significantly (*P* > 0.05), both FMT1 and FMT2 were significantly separated from CON (*P* < 0.05). By D 7, while CON and FMT1 showed no significant separation (*P* > 0.05), FMT2 and FMT3 were markedly distinct from CON (*P* < 0.05). On D 14, all FMT groups exhibited significant separation from CON (*P* < 0.05). Specifically, 42 pathways were significantly different among the groups on D 5 (Supplementary file 1, Table S5.1), while 30 pathways showed significant changes on both D 7 (Supplementary 1, Table S6.1) and D 14 (Supplementary 1, Table S7.1). Notably, on D 5 (Supplementary file 1, Table S5.2), FMT1 was significantly enriched in amino acid biosynthesis and carbohydrate degradation compared to CON, while all FMT groups showed significant enrichment in nucleoside and nucleotide biosynthesis (*P* < 0.05). By D 7 (Supplementary file 1, Table S6.2), FMT2 and FMT3 were enriched in nucleoside and nucleotide biosynthesis, with FMT2 additionally displaying significant enrichment in cofactor, prosthetic group, electron carrier, and vitamin biosynthesis, fatty acid and lipid biosynthesis, and carbohydrate biosynthesis pathways (*P* < 0.05). Meanwhile, FMT1 and FMT3 were enriched in carbohydrate degradation (*P* < 0.05). On D 14 (Supplementary file 1, Table S7.2), FMT2 and FMT3 remained enriched in cofactor, prosthetic group, electron carrier, and vitamin biosynthesis, as well as in the TCA cycle and inorganic nutrient metabolism, while all FMT groups showed significant enrichment in carbohydrate biosynthesis compared to the CON group (*P* < 0.05).

### FMT alters recipients’ ability to ferment inulin and citrus pectin

**Fig. 6:**
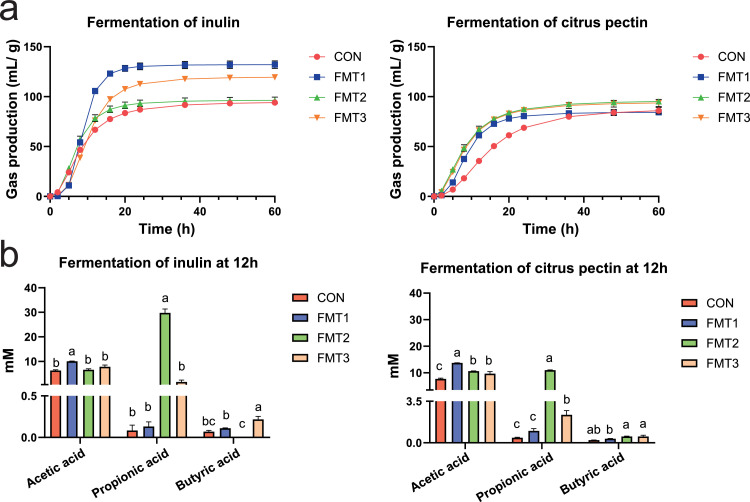
*In vitro* fermentation profiles of FMT recipient microbiota. **(a)** Fitted cumulative gas production during fermentation of inulin and citrus pectin by recipient microbiota (mL/g). **(b)** Short chain fatty acid production after 12 h fermentation (mM). FMT1, FMT2, and FMT3 received inocula from broilers, laying hens, and broiler breeders, respectively. Incubation temperature during fermentation: 41°C. Data represent mean values ± SEM. *n* = 3 technical replicates. Statistical analyses: Kruskal-Wallis test with Dunn’s post hoc test for short chain fatty acid production.

The fitted cumulative gas production curve of recipients is shown in Fig. 6a. The kinetic parameters of inulin fermentation by different FMT recipients *in vitro* are presented in Table 3. For the inulin fermentation, compared to the CON, the FMT1 and FMT3 exhibited a higher gas production (A, *P* < 0.001). The time to reach half of asymptote (B) was significantly longer in FMT3 and shorter in FMT2 compared to CON (*P* < 0.001). The maximum rate of gas production (R_max_) was significantly higher in FMT1 compared to CON (*P* < 0.001). The time to reach R_max_ (T_max_) was significantly longer in FMT1 and FMT3 compared to CON (*P* < 0.001). For citrus pectin fermentation, there were no differences between CON and all FMT groups in A (*P* > 0.05). All FMTs groups exhibited shorter B and T_max_, and higher R_max_ compared to CON (*P* < 0.001).

**Table 3:** *In vitro* fermentation kinetics of inulin and citrus pectin by recipient gut microbiota.

Fermentation of inulin					
	A (mL/g DM)	B (h)	R_max_ (mL/g DM/h)	T_max_ (h)	R^2^
CON	95.23^c^	8.17^b^	8.02^b^	5.22^b^	0.9976
FMT1	132.08^a^	8.71^b^	17.41^a^	7.80^a^	0.9896
FMT2	96.61^c^	6.97^c^	10.77^b^	5.22^b^	0.9902
FMT3	119.85^b^	10.10^a^	10.48^b^	8.22^a^	0.9944
SEM	4.85	0.35	1.08	0.43	
*P* value	<0.001	<0.001	<0.001	<0.001	
Fermentation of citrus pectin					
CON	88.82	14.20^a^	4.46^b^	9.73^a^	0.9755
FMT1	84.56	8.64^b^	8.13^a^	6.80^b^	0.9943
FMT2	96.79	8.00^b^	7.96^a^	4.71^c^	0.9956
FMT3	94.95	8.14^b^	7.90^a^	5.13^bc^	0.9866
SEM	1.94	0.8	0.48	0.62	
*P* value	0.065	<0.001	<0.001	<0.001	

Note: The different letters in a row mean a significant difference (*P* < 0.05). *n* = 3 technical replicates. A: the total gas production. B: the time to half asymptote. R_max_: the maximum rate of gas production. T_max_: the time at which the maximum rate of gas production is reached. DM: dry matter. SEM: Standard error of the mean. R^2^: coefficient of determination.

As shown in Fig. 6b, in the *in vitro* fermentation of inulin, FMT1 produced highest acetic acid while FMT2 produced highest propionic acid (*P* < 0.05), and FMT3 produced highest butyric acid when compared to CON (*P* < 0.05). In the *in vitro* fermentation of citrus pectin, all FMT groups produced a higher amount of acetic acid than CON (*P* < 0.05). FMT2 and FMT3 produced higher amounts of propionic acid than CON (*P* < 0.05).

## Discussion

We first evaluated the fecal microbial composition of different donor chickens. Analysis revealed three distinct microbial patterns among the donors, reflecting differences in age, physiological status, diet, and genetic background (Dai et al., 2022; Li et al., 2022; Rychlik, 2020). Results indicated that the fecal microbiota from the broiler donors was predominantly composed of Firmicutes and Actinobacteriota, while that from the laying hen donors was mainly dominated by Firmicutes, Bacteroidota, and Proteobacteria, which is consistent with previous studies (Li et al., 2022; Zhu et al., 2019). Some studies have compared unique operational taxonomic units (**OTUs**) between broiler and laying hen feces, showing that broiler-specific OTUs typically belong to Firmicutes, while laying hen-specific OTUs are primarily Bacteroidota (Videnska et al., 2014). Bacteroidota are known for their ability to degrade complex carbohydrates (McKee et al., 2021), suggesting differences in metabolic capacity for complex carbohydrates between broilers and laying hens. The microbial composition of broiler breeders’ feces differed from both donor groups, with a higher abundance of Actinobacteriota than Firmicutes, a change presumably driven by age (Dai et al., 2022).

Differences in donor fecal microbial composition highlight the possibility of metabolic differences of the microbiota (Logtenberg et al., 2020). Subsequently, we fermented inulin and citrus pectin using fecal microbiota from these different donors. The lower fermentation rates for both inulin and citrus pectin observed in the broiler donors’ inoculum, as evidenced by lower R_max_, and higher B and T_max_ values, is in line with a lower microbial diversity and functional maturity, resulting in reduced fermentation capacity (Dai et al., 2022; Rychlik, 2020). The faster fermentation capacity of the laying hen donors’ inoculum is due to the presence of more complex and mature fiber-degrading networks in their microbial communities (Dai et al., 2022).

SCFA are end products of microbial fermentation (Mahmood and Guo, 2020). The higher SCFA concentration, particularly propionic acid, in laying hen donors’ *in vitro* fermentation maybe linked to the presence of *Bacteroides*. This is because many *Bacteroides* species are reported to possess enzymes involved in propionic acid–producing pathways, and some also encode xylose isomerases and other enzymes required for polysaccharide degradation. (Fan et al., 2023; Ndeh et al., 2017; Ondrej et al., 2016). Interestingly, during inulin fermentation, the abundance of *Bacteroides* in the broiler breeders’ inoculum was higher than in the one of laying hens, which was further confirmed by LEfSe analysis. This result contrasts with the microbial composition in fecal samples, suggesting that broiler breeder microbiota harbor *Bacteroides* populations with greater adaptability or competitiveness, granting them an advantage in utilizing simple type of fiber (Ma et al., 2020). In contrast, *Bacteroides* abundance did not increase during inulin fermentation in the laying hens’ inoculum, likely due to inulin’s low specificity as a fiber degradable by multiple bacterial taxa (Cantu-Jungles and Hamaker, 2020). This probably limits *Bacteroides* dominance, as their microbial communities may be less optimized for this substrate and the microbial composition shifts towards other genera (Medvecky et al., 2018). For instance, *Subdoligranulum*, which is known to possess enzymes involved in butyric acid production (Ondrej et al., 2016), may contribute to the elevated levels of butyric acid observed in the laying hen fermentation broth. Additionally, *Bifidobacterium*, which has been reported to synthesize acetic acid via the acetyl-CoA and Wood-Ljungdahl pathways, was enriched in laying hen inulin fermentations (Liu et al., 2021; Ragsdale and Pierce, 2008). The higher SCFA production in laying hen fermentations was also associated with the abundance of *Collinsella* , consistent with previous reports (Lammert et al., 2021). For structurally complex citrus pectin (Wu et al., 2021), the *Bacteroides* abundance in fermentation broths was similar between laying hens and broiler breeders. The inherent complexity of citrus pectin balanced microbial competitive advantages, leading to outcomes distinct from those observed with inulin (van Trijp et al., 2020). Furthermore, *Clostridium* and *Bifidobacterium* known for pectin degradation (van Trijp et al., 2020) were enriched in the broiler citrus pectin fermentation. Functional prediction revealed donor-specific metabolic strategies, defined as the distinct metabolic pathway preferences shaped by donor and substrate. During fermentation, the broiler donors’ inoculum exhibited predicted enrichment of carbohydrate degradation associated with monosaccharides during both inulin and citrus pectin fermentation, reflecting microbial adaptation for rapid energy extraction (Rychlik, 2020; Xia et al., 2021). In contrast, the donor inocula from laying hen and broiler breeder exhibited predicted enrichment of amino acid biosynthesis, fatty acid and lipid biosynthesis, and carbohydrate biosynthesis pathways. These predicted functional profiles suggest a broader metabolic potential that may enable a more diverse metabolite production (Bernard et al., 2024; Rychlik, 2020). In summary, fiber degradation involves substrate-driven microbial adaptations and interactions, with the shift depending on the initial microbial composition (Cantu-Jungles and Hamaker, 2020).

Next, we investigated how these donor-specific microbial communities establish themselves in recipient birds and influence fiber degradation. While FMT has been frequently reported to enhance cecal microbial diversity in broilers (Gong et al., 2019; Ma et al., 2023), some studies suggest that FMT may lead to a more uniform microbial composition in recipients, reducing alpha diversity (Elokil et al., 2022). In our experiment, the impact of FMT on alpha diversity varied depending on the microbiota source. FMT2 consistently increased alpha diversity at all time points, whereas FMT1 and FMT3 had limited effects on the Shannon index, suggesting that the recipient’s microbiota was dominated by a few highly abundant species, resulting in a less even community structure (Finotello et al., 2018). Beta diversity in this study corroborated the results of previous studies (Song et al., 2024; Yan et al., 2021), indicating that all the FMT groups altered the structural composition of the recipients’ microbiota (Finotello et al., 2018).

In this study, we observed that early microbiota transplantation significantly shifted the dominant phyla in recipient broilers. A high Firmicutes/Bacteroidota (F/B) ratio is commonly observed in large-scale commercial broiler production, reflecting management practices optimized for rapid growth but often at the expense of gut health (Mancabelli et al., 2016), which aligns with the elevated F/B ratio in the CON and FMT1 groups (broiler donors) compared to the lower ratios in FMT2 and FMT3. Notably, the elevated Bacteroidota levels in FMT2 and FMT3 recipients aligned with donor microbiota profiles, suggesting that FMT may enhance the recipients’ capacity to utilize complex carbohydrates (McKee et al., 2021). Furthermore, Actinobacteriota increased while Proteobacteria decreased across FMT groups, underscoring the potential role of FMT in promoting gut health and reducing pathogen colonization (Binda et al., 2018; Dai et al., 2018; Hooper and Macpherson, 2010). At the genus level, FMT recipients exhibited higher abundances of *Rikenellaceae_RC9_gut_group, Bacteroides, Faecalibacterium, Mucispirillum*, and *Muribaculaceae*, which are involved in immune modulation, gut homeostasis maintenance, and nutrient metabolism (Hwang et al., 2023; Macia et al., 2015; Martín et al., 2023; Parada Venegas et al., 2019; Zafar and Saier Jr, 2021), though the patterns among FMT groups varied. It is worth noting that *Escherichia–Shigella* was significantly enriched in the CON group. This genus is composed of many pathogen strains, such as *E. coli* O157:H7, which are capable of intestinal epithelial invasion and intestinal barrier disruption, ultimately triggering immune stress (Gong et al., 2019; Shi et al., 2014). *Escherichia–Shigella* are mainly present in newly hatched chicks and is inversely related to age. A reduction in their abundance indicates microbiota maturation and compositional stability (Akram et al., 2024; Gong et al., 2019; Jurburg et al., 2019). This finding indicates that FMT may mitigate early colonization by this high-risk genus, potentially reducing downstream health risks.

Studies have reported that FMT significantly increases the concentrations of acetic acid (Gong et al., 2019), propionic acid (Gong et al., 2019; Marcolla et al., 2023; Metzler-Zebeli et al., 2019), and butyric acid (Gong et al., 2019) in the cecal content of recipients, which is consistent with our research. However, we observed donor-specific SCFA production patterns in our experiment. The FMT1 group exhibited a significant increase in butyric acid during the first week, likely driven by the enrichment of *Faecalibacterium* and *Subdoligranulum*. These genera are known to possess key enzymes for butyrate synthesis, including acetyl-CoA acetyltransferase, 3-hydroxyacyl-CoA dehydrogenase, and enoyl-CoA hydratase (Ondrej et al., 2016). Both FMT2 and FMT3 groups showed marked increases in propionic acid and valeric acid, correlating with higher abundances of propionate-producing bacteria such as *Bacteroides* (Pandit et al., 2018). Notably, this observation is consistent with our *in vitro* findings, suggesting that the propionate-producing potential of laying hen-derived microbiota may be reflected *in vivo*. Branched-chain fatty acids (**BCFAs**), such as iso-butyric and iso-valeric acids, were significantly elevated in the FMT groups *in vivo*. These metabolites are typically derived from the deamination and decarboxylation of branched-chain amino acids via microbial proteolytic fermentation, in which genera such as *Bacillus* may participate through the branched-chain keto acid dehydrogenase complex (Oliphant and Allen-Vercoe, 2019). When carbohydrate substrates become limited, certain gut microbes preferentially switch from fermenting carbohydrates to breaking down residual proteins, thereby shifting fermentation from saccharolytic to proteolytic pathways (Singh and Kim, 2021). This proteolytic fermentation can be associated with the generation of potentially harmful nitrogenous metabolites that impair gut barrier function and overall intestinal health (Liu et al., 2021). However, BCFAs have also been reported to exert beneficial effects, such as inhibiting *Clostridium difficile* growth (McDonald et al., 2018), regulating hepatic glucose and lipid metabolism (Heimann et al., 2016), and serving as alternative energy sources for enterocytes during butyric acid deficiency (Jaskiewicz et al., 1996). These potential benefits remain speculative without direct evidence in the present study. Future work should include measurements of proteolytic enzyme activities or ammonia concentrations to clarify the extent of proteolytic fermentation and its potential consequences.

Over time, the predicted functional differences of the microbiota between the FMT and CON groups became more pronounced, likely due to the successful establishment of a cecal microbial niche by FMT (Walter et al., 2018). The pioneering microorganisms that first colonize these niches can engage in competitive, antagonistic, or mutualistic interactions with other community members, thereby selectively altering their abundance and functions (Walter et al., 2018). By D 14, the FMT groups showed significant enrichment in predicted pathways related to ‘cofactor, prosthetic group, electron carrier, and vitamin biosynthesis’, as well as the ‘TCA cycle’. The first predicted pathway involves the production of coenzymes and B vitamins, which are key cofactors in fiber degradation (LeBlanc et al., 2017). The TCA cycle, as the core of cellular energy metabolism, utilizes fiber degradation products to generate energy for the host (LeBlanc et al., 2017). Under anaerobic conditions, some microbes cannot complete a full TCA cycle and instead produce succinate as an intermediate metabolite (Fernández-Veledo and Vendrell, 2019; Spiga et al., 2017). Notably, certain Bacteroidota members ferment pentose and hexose carbohydrates through the succinate pathway to generate propionate (Fernández-Veledo and Vendrell, 2019; Louis and Flint, 2017), which explains the increased propionate levels observed in the FMT groups in our study.

In the *in vitro* fermentation experiments using recipient microbiota, donor-specific fermentation patterns were partially observed in recipients, varying significantly depending on substrate and donor type. Both FMT1 and FMT3 significantly increased the maximum gas production during inulin fermentation, consistent with the trends observed in donor *in vitro* fermentation, suggesting successful colonization and functional establishment of microbial populations, potentially driven by *Bacteroides, Faecalibacterium* and *Subdoligranulum*, which exhibit effective utilization of simple fibers (Cantu-Jungles and Hamaker, 2020; Ma et al., 2020). However, no changes in gas production were detected during citrus pectin fermentation, likely due to the substrate’s structural complexity presenting metabolic challenges for microbiota, as pectin degradation typically requires specialized enzymes (van Trijp et al., 2020). Notably, FMT consistently enhanced fermentation rates across substrate types, likely due to introduced microbial communities promoting synergistic interaction, thereby improving substrate utilization efficiency (Flint et al., 2012). For example, some members from *Bacteroides*, which express elevated levels of CAZymes, may contribute to the fermentation efficiency (Wardman et al., 2022). Interestingly, the FMT groups maintained consistent SCFA production patterns with both *in vitro* donor and *in vivo* recipient results, particularly in elevated propionate levels. Propionic acid production primarily occurs through the succinate pathway, mainly found in Bacteroidota and Negativicutes class Firmicutes, with an alternative route being the propanediol pathway used by *Lachnospiraceae* and *Blautia* (Louis and Flint, 2017). Given that unclassified *Lachnospiraceae* and *Blautia* were less abundant in FMT groups in our study, we speculate that the FMT-induced propionate increase is associated with the succinate pathway. However, a comprehensive understanding of the underlying mechanisms will require further investigation using culturomics, metagenomic, and metabolomic approaches.

This study has some limitations that should be acknowledged. First, as the FMT experiment was done using pooled fecal samples, the *in vitro* fermentations were conducted with these pooled inocula. This may not capture inter-individual variation. Our aim was to create three donors with a very distinct microbiota to investigate the importance of the donor microbial composition. To enable a firm statement on the ideal donor (e.g. breed, age), future studies can consider individual donors as inocula for both the *in vivo* and *in vitro* experiment, resulting also for the latter in multiple biological repetitions rather than the technical repetitions we obtained now. As some studies have shown that chickens raised under different rearing conditions exhibit substantial microbiota differences (Rychlik, 2020), these differences are likely much larger than the inter-individual variation within a single rearing condition (Ding et al., 2017). Nevertheless, further studies are needed to validate these fermentation characteristics using individual inocula. Second, it should be noted that the present study followed recipients only until D 14. The first two weeks are considered a critical window for microbial establishment in broilers (Zhao et al., 2025), and early microbial modulation has been reported to exert effects that persist up to D 35 (Rubio, 2019). Nevertheless, it is important for future studies to extend the observation period to confirm these effects. Third, the functional profiling in this study relied on PICRUSt2 predictions based on 16S rRNA gene data, which is only predictive. Future research should employ shotgun metagenomics to corroborate the predicted functional potential observed here. Finally, no targeted pathogen screening such as Salmonella, *Clostridium perfringens*, or pathogenic *Escherichia coli* was performed on the donor material. In addition, potential reductions in microbial viability during freeze–thaw of donor inocula should be considered. Future studies aiming at practical application of FMT should incorporate appropriate microbiological safety assessments, such as plating or molecular detection of major poultry pathogens, and monitor the effects of storage and thawing of the donor’s feces on microbial viability.

## Conclusion

In poultry diets, soluble fibers like inulin and citrus pectin, found in plant-based feed ingredients or byproducts, are increasingly relevant for promoting gut health and feed efficiency. Our study demonstrates that distinct microbial compositions, present in different chicken donors led to differential fermentative capacities for both simple (inulin) and complex (citrus pectin) fiber. Through FMT, we successfully altered the recipient’s microbial structure up to D 14, enhancing VFA production. Microbial functional predictions revealed that FMT recipients developed broader metabolic diversity. Subsequent *in vitro* fermentation assays further elucidated how donor-derived microbial communities shape divergent fiber degradation kinetics in recipients in a substrate-dependent manner. These findings suggest that FMT may enhance fiber degradation in broilers by leveraging donor-specific fermentative ability, providing a foundation for further studies on microbiota-targeted strategies to promote gut microbial function.

## Funding

This work was supported by China Scholarship Council (CSC) of the Ministry of Education, P.R. China (CSC No. 202206850006).

## Data availability

The 16S rRNA gene sequencing data can be accessed under Bioproject accession PRJNA1299803 at the NCBI website. The raw data of both *in vitro* donor and *in vitro* recipient fermentation are placed in Supplementary file 1 (Table S8, S9, S10, S11). The raw data VFA concentrations are also present in Supplementary file 1 (Table S12, S13, S14).

## CRediT authorship contribution statement

**Haoran Zhao:** Writing – review & editing, Writing – original draft, Methodology, Investigation, Funding acquisition, Formal analysis, Conceptualization. **Muhammad Zeeshan Akram:** Writing – review & editing, Methodology, Investigation. **Luke Comer:** Writing – review & editing, Methodology, Investigation. **Matthias Corion:** Writing – review & editing. **Elena Fako:** Writing – review & editing. **Nadia Everaert:** Writing – review & editing, Supervision, Project administration, Funding acquisition, Conceptualization.

## Disclosures

Haoran Zhao reports financial support was provided by China Scholarship Council. If there are other authors, they declare that they have no known competing financial interests or personal relationships that could have appeared to influence the work reported in this paper.

## Appendix Supplementary materials

Image, application 1

Image, application 2
