# Supplementary material for: Early life fecal microbiota transplantation enhances fermentation potential by changing the microbial profiles in broiler chickens
Source: Poult Sci. 2025 Dec 2;105(1):106189. doi: 10.1016/j.psj.2025.106189 (PMC12757631; doi:10.1016/j.psj.2025.106189)
Supplement: Supplementary file 2 [file mmc2.docx]

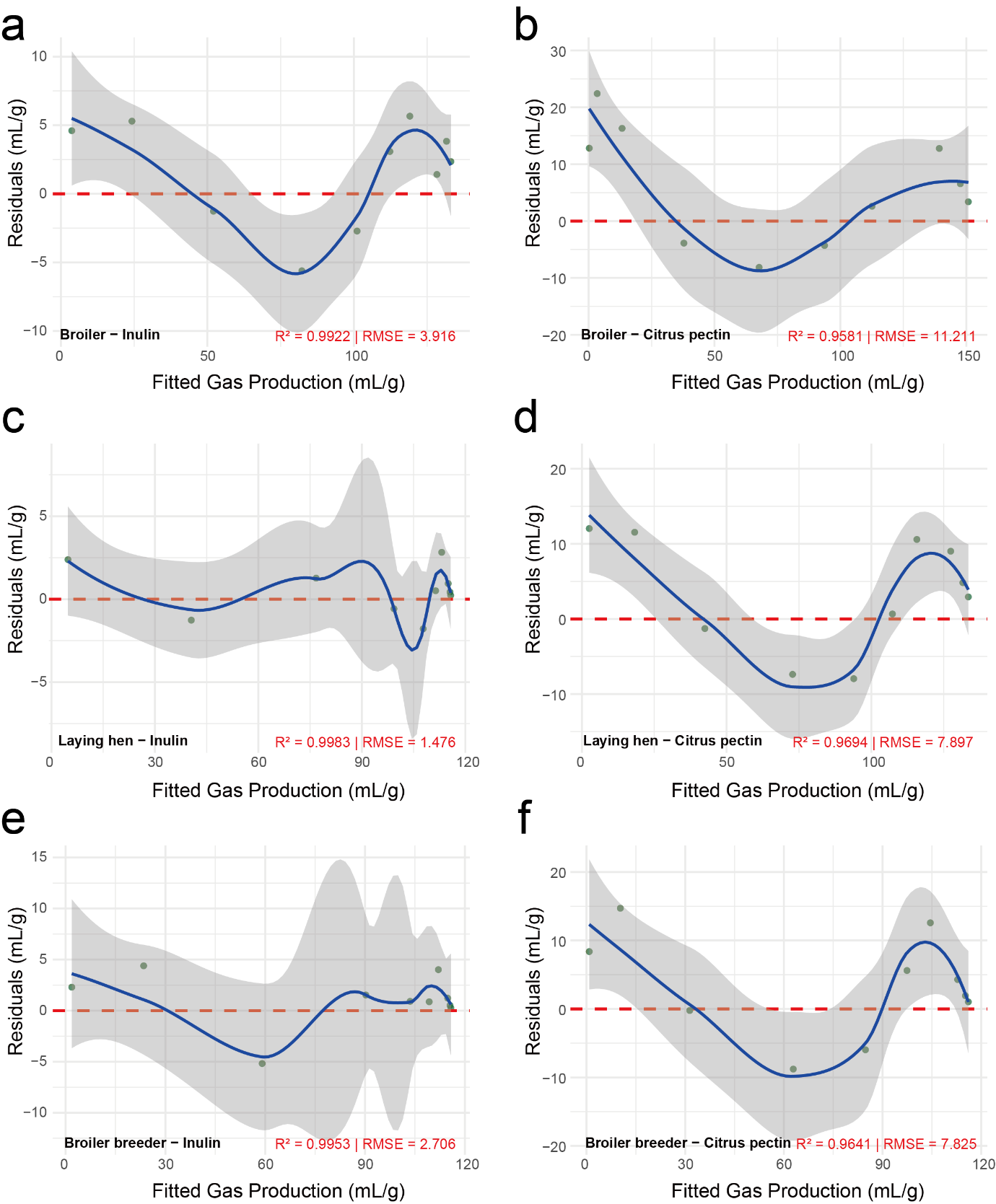


Fig. S1. Residual diagnostics of the Groot monophasic model for *in vitro* fermentation by donor microbiota. Residual plots showing the differences between observed and predicted gas production values for: (a) broiler microbiota fermenting inulin, (b) broiler microbiota fermenting citrus pectin, (c) laying hen microbiota fermenting inulin, (d) laying hen microbiota fermenting citrus pectin, (e) broiler breeder microbiota fermenting inulin, (f) broiler breeder microbiota fermenting citrus pectin. The blue line represents the locally estimated scatterplot smoothing (**LOESS**) trend, and the red dashed line indicates the zero-residual reference. The coefficient of determination (**R²**) and root mean square error (**RMSE**) quantify model performance for each condition.


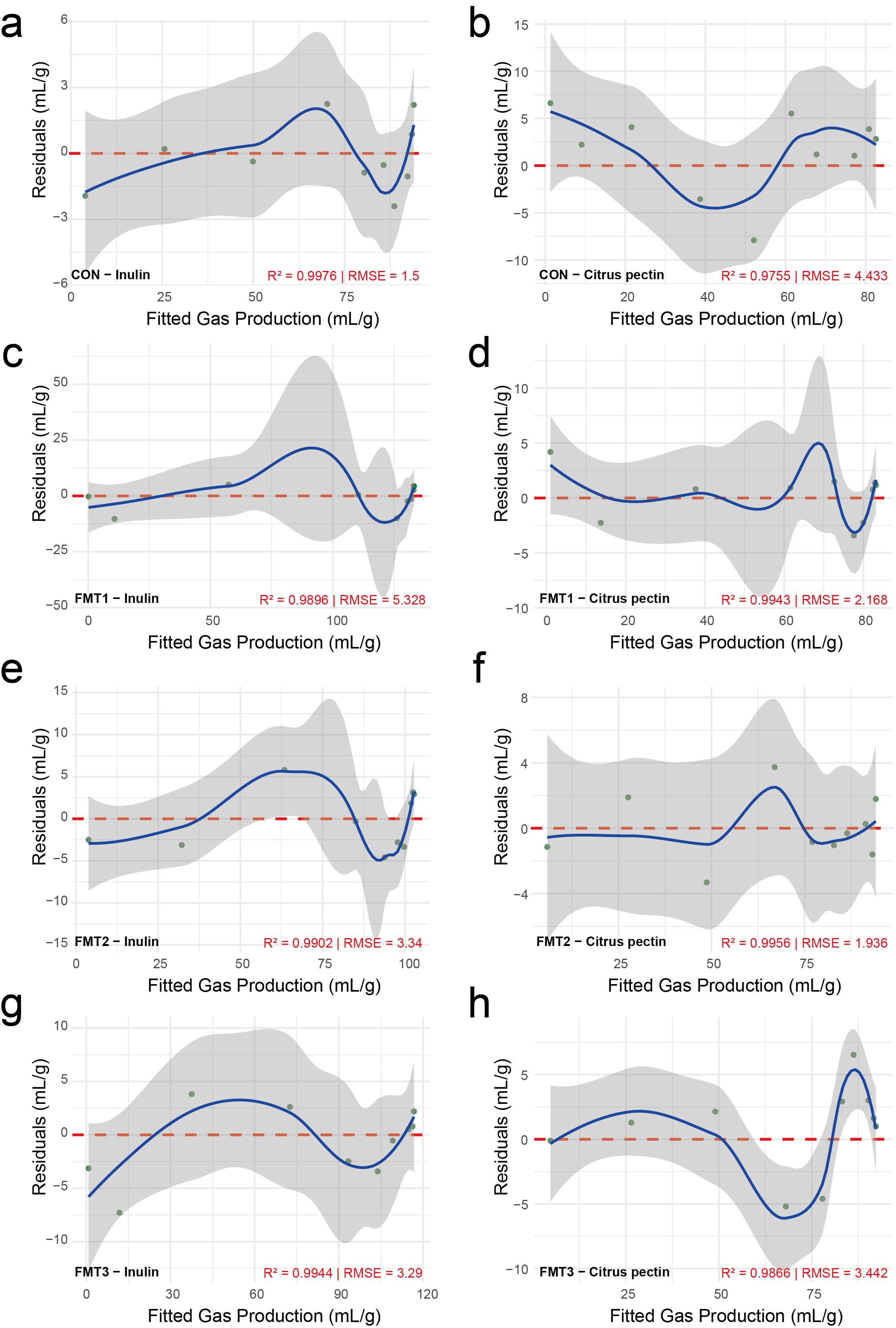


Fig. S2. Residual diagnostics of the Groot monophasic model for *in vitro* fermentation by microbiota from recipient cecal content. Residual plots showing the differences between observed and predicted gas production values for: (a) CON fermenting inulin, (b) CON fermenting citrus pectin, (c) FMT1 fermenting inulin, (d) FMT1 fermenting citrus pectin, (e) FMT2 fermenting inulin, (f) FMT2 fermenting citrus pectin, (g) FMT3 fermenting inulin, (h) FMT3 fermenting citrus pectin. The blue line represents the locally estimated scatterplot smoothing (**LOESS**) trend, and the red dashed line indicates the zero-residual reference. The coefficient of determination (**R²**) and root mean square error (**RMSE**) quantify model performance for each condition. FMT1, FMT2, and FMT3 received inocula from broilers, laying hens, and broiler breeders, respectively.


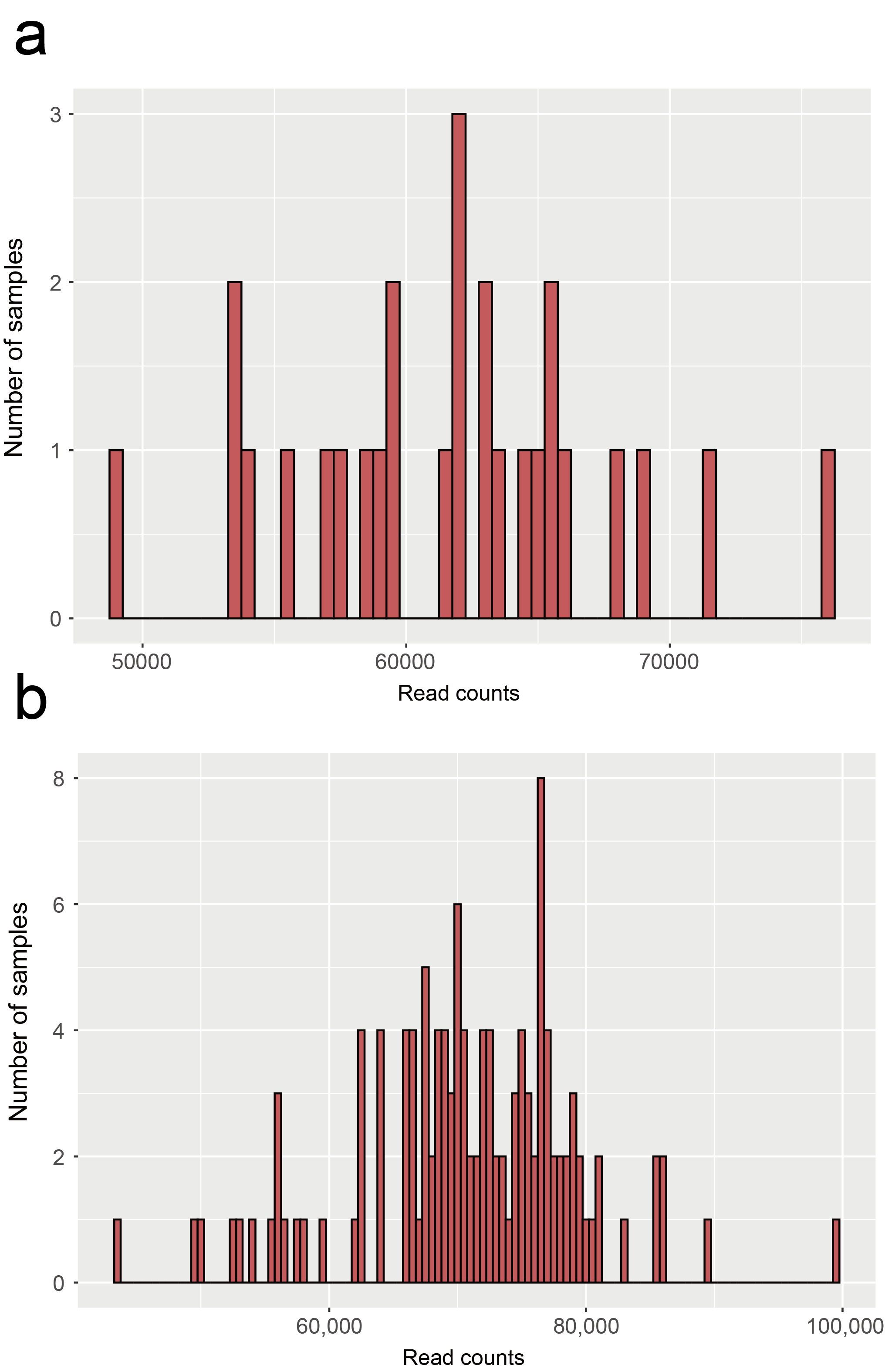


Fig. S3. Distribution of sequence depth. (a) Sequence depth distribution for donor microbiota during *in vitro* fermentation. (b) Sequence depth distribution in recipient cecal microbiota.


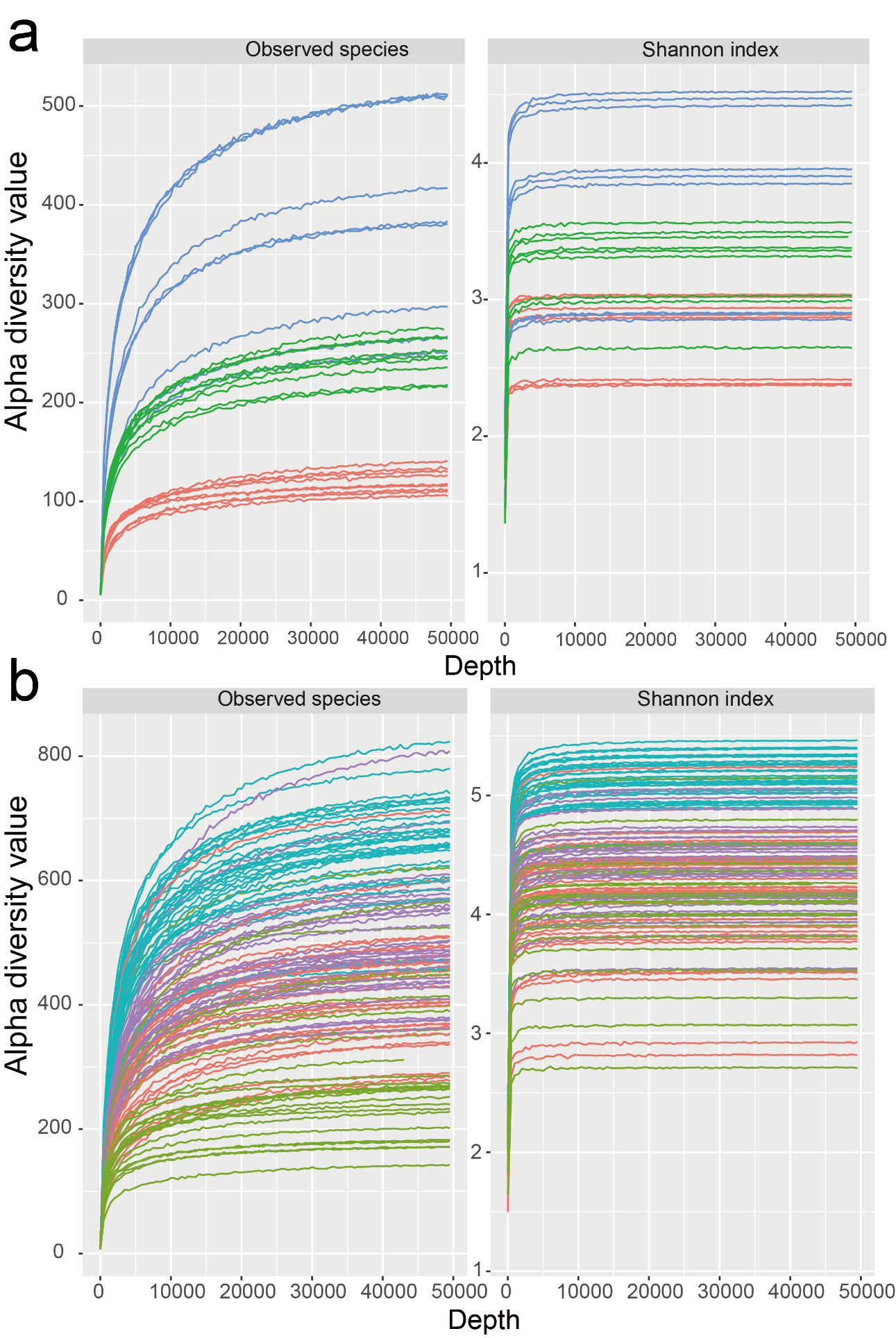


Fig. S4. Rarefaction curves of alpha diversity. (a) Rarefaction curves of alpha diversity for donor microbiota during *in vitro* fermentation. (b) Rarefaction curves of alpha diversity for the cecal content of recipients. Each line represents an individual sample.
